# Effectiveness of Antipsychotics in Reducing Suicidal Ideation: Possible Physiologic Mechanisms

**DOI:** 10.3390/healthcare9040389

**Published:** 2021-04-01

**Authors:** Ahmed Hassan, Vincenzo De Luca, Nasia Dai, Alessio Asmundo, Nunzio Di Nunno, Marcellino Monda, Ines Villano

**Affiliations:** 1 Group for Suicide Studies, CAMH, Department of Psychiatry, University of Toronto, Toronto, ON M5T1R8, Canada; Ahmed.hassan@camh.ca (A.H.); Nasia.dai@camh.ca (N.D.); 2 Department of Pharmacology and Toxicology, University of Toronto, Toronto, ON M5T1R8, Canada; 3 Department of Biomedical and Dental Sciences, and of Morphological and Functional Images, Section of Legal Medicine, University of Messina, 98122 Messina, Italy; alessio.asmundo@unime.it; 4 Department of History, Society and Studies on Humanity, University of Salento, 73100 Lecce, Italy; nunzio.dinunno@unisalento.it; 5 Department of Experimental Medicine, Universita’ della Campania ‘Luigi Vanvitelli’, Via Santa Maria a Costantinopoli 16, I-80138 Naples, Italy; Marcellino.monda@unicampania.it (M.M.); ines.villano@unicampania.it (I.V.)

**Keywords:** schizophrenia, suicide, antipsychotics, clozapine, Beck Scale for Suicidal Ideation

## Abstract

*Background*: The aim of this study is to evaluate whether any specific antipsychotic regimen or dosage is effective in managing suicidal ideation in schizophrenia. Four comparisons were conducted between: (1) clozapine and other antipsychotics; (2) long-acting injectable and oral antipsychotics; (3) atypical and typical antipsychotics; (4) antipsychotics augmented with antidepressants and antipsychotic treatment without antidepressant augmentation. *Methods*: We recruited 103 participants diagnosed with schizophrenia spectrum disorders. Participants were followed for at least six months. The Beck Scale for Suicidal Ideation (BSS) was used to assess the severity of suicidal ideation at each visit. We performed a multiple linear regression model controlling for BSS score at study entry and other confounding variables to predict the change in the BSS scores between two visits. *Results*: Overall, there were 28 subjects treated with clozapine (27.2%), and 21 subjects with depot antipsychotics (20.4%). In our sample, 30 subjects experienced some suicidal ideation at study entry. When considering the entire sample, there was a statistically significant decrease in suicidal ideation severity in the follow-up visit compared to the study entry visit (*p* = 0.043). *Conclusions*: To conclude, our preliminary analysis implies that antipsychotics are effective in controlling suicidal ideation in schizophrenia patients, but no difference was found among alternative antipsychotics’ classes or dosages.

## 1. Background

Suicide in schizophrenia is a major public health problem. Longitudinal studies in schizophrenia patients showed a suicide rate of approximately five percent [1,2]. Suicidal attempts occur at a much higher rate, ranging from 30% to 49% in some studies [3,4,5,6]. Furthermore, suicidal ideation tends to rapidly progress, regardless of the mood status, to actual attempts and many studies confirmed a positive correlation between suicidal ideations and suicidal behaviors [2,7]. In a large cross-sectional study, participants who had psychosis and reported suicidal ideation were 3.5 times at higher risk to attempt suicide compared to people with suicidal ideation but without psychosis [8].

In schizophrenia, several demographic and clinical features seem to be associated with suicide such as male gender, young age, higher level of education, presence of depressive symptoms, past suicide attempts and family history of suicide [6,9,10,11,12,13,14,15].

Various investigations have been conducted to determine if certain pharmacological agents can control suicidal thoughts in patients with schizophrenia. In a cohort study conducted by Tiihonen and colleagues, of the 2230 patients with schizophrenia, 26 suicides occurred in patients not taking antipsychotics compared to only one suicide from patients on antipsychotics [16]. Ward and colleagues conducted a retrospective study and indicated that schizophrenia patients with good compliance have reduced suicidal risk, but this was not consistent among all the study participants [17]. Though the majority of studies concluded a positive effect of antipsychotics in reducing suicide risk, Ran and colleagues found no statistically significant difference in suicidal rate between treated and non-treated patients with schizophrenia [18].

A prospective study found that discontinuation of long-acting injectable medications for patients with chronic schizophrenia resulted in more self-injurious behavior [19]. This may indicate that long-acting injectable antipsychotics that ensure adherence, could be a better option for some suicidal patients. The practice of augmenting antipsychotic treatment with antidepressants has moderate-to-good evidence for effectiveness in treating depressive symptoms in schizophrenia patients [20]. In a randomized placebo-controlled trial (RCT), citalopram was more effective than placebo for lowering suicidal ideation in psychotic patients after 12 weeks of treatment [21]. However, citalopram was no different than placebo for those without suicidal ideation at baseline in avoiding the onset of emergent suicidal ideation [21]. Current research has, therefore, suggested that antipsychotic treatment and the use of long-acting injectable medications may be beneficial in suicidal patients with psychoses. As a result, schizophrenia patients treated with antipsychotics, may have reduced risk of suicide in comparison to patients who are not taking antipsychotics.

Treatment with atypical antipsychotics showed overall benefits in terms of side effects but superiority of atypical antipsychotics over typical antipsychotic on functional outcome is questionable [22]. The FDA approved clozapine for reducing suicidal thoughts in schizophrenia based on studies that reported that clozapine has the ability to reduce suicidal ideation compared to other antipsychotics [23,24,25,26]. Most of these studies were randomized controlled trials in treatment-resistant patients. A cohort study conducted by De Hert and colleagues followed patients for 11 years and found a significant reduction of mortality for clozapine over perphenazine [27]. However, this study presented some methodological problems including the lack of control for confounding factors [27]. In comparison, a naturalistic study by Sernyak and colleagues did not find clozapine any different from other antipsychotics in reducing suicidal rates especially after short-term exposure [28]. Current research has therefore yet to confirm if a specific type/class of antipsychotic or augmenting medication can reduce suicidal ideation in patients with schizophrenia.

The aim of this naturalistic cohort study is to evaluate if any particular type, or regimen of antipsychotics is effective in managing suicidal thoughts in schizophrenia patients after an observation time of at least six months of antipsychotic treatment. We conducted four medication regimen comparisons. The first analysis compared the change in suicidal ideation between clozapine-treated patients and those treated with other types of antipsychotics. The second comparison was between long-acting injectable and oral antipsychotics. The third one was between atypical antipsychotics, including clozapine, and typical antipsychotics. The last comparison was between patients on antipsychotics augmented with antidepressants and patients treated with antipsychotics without antidepressants.

## 2. Methods

### 2.1. Study Design

This is a naturalistic, prospective cohort study in which we assessed participants at two-time points. The first assessment was at study entry and the second assessment was anytime six months after the initial assessment. The baseline was considered the assessment at study entry regardless of the stage of treatment or the duration of illness. They were assessed using a semi-structured interview at study entry and follow-up. Follow up visits were at least six months after the initial assessment and were considered different than the clinical visit with the treating team. These were follow-up visits that were convenient for the participant. All participants were treated with antipsychotics. The change in suicidal thoughts severity between the two visits was the main outcome. Socio-demographic characteristics and medication details were collected from both the participants’ report and their electronic medical records. We used the Defined Daily Doses (DDD), developed by the WHO, to measure the equivalent daily dose of antipsychotic for each participant [29].

We also standardized the change of each prescribed antipsychotic dose as chlorpromazine equivalent (CPZe) [30]. We defined polypharmacy as the prescription of two or more antipsychotics at the same time. The study was approved by the CAMH REB and all participants signed an informed consent form to participate in the study.

### 2.2. Patient Selection

Patients diagnosed with schizophrenia spectrum disorders were consecutively recruited from the Centre for Addiction and Mental Health (CAMH), a Canadian teaching hospital located in Toronto, Ontario. All study participants were seen in the hospital outpatient clinics. Participants were included if they were older than 18, had a diagnosis of schizophrenia or schizoaffective disorder, and were fluent in English. Participants with psychosis due to traumatic brain injury or general medical condition were excluded. The participants were recruited for this study between 2012 and 2014.

### 2.3. Measures

The Structured Clinical Interview for DSM-IV (SCID) was used to confirm the diagnosis of schizophrenia spectrum disorders [31]. Lifetime DSM-IV diagnoses of alcohol or drug use disorders were assessed at study entry. Beck Scale for Suicidal Ideation (BSS), a self-reported scale was used—was used to measure to assess the severity of suicidal ideation and confirm its presence at the follow-up visit [32]. The BSS is a 19-item questionnaire and each item assess the intensity of suicidal ideation. Scores ranges from 0 to 38 with higher scores indicating higher suicide risk. The scale includes the assessment of wish to live/die, reasons for living/dying, desire to make a suicidal attempt, duration/frequency of suicidal ideation, control of suicidal action, deterrents to active attempt, attitude toward ideation, expectation of an actual attempt and its preparation. For the analysis, antipsychotics were classified as oral or long-acting injectable (type), and as a typical or atypical (class).

### 2.4. Statistical Analysis

All statistics were conducted using R version 3.1.2. Initially, a paired t-test was conducted to compare the effect of all types of antipsychotic regimen on suicidal ideation assessed using the BSS at study entry and follow-up. A correlation test was performed between the BSS change between the two visits and the baseline BSS score. Additionally, the correlation between BSS change and antipsychotic dosage was tested. The clinical-demographic characteristics were compared between the different antipsychotic regimen by independent t-test or chi-square when appropriate. An independent t-test was used to compare the difference of BSS score between the two visits. BSS changes were incorporated in a linear regression model to test the effect of the different antipsychotic regimens. Linear regression controlled for age, education, smoking status, polypharmacy, BSS score at study entry and the interval duration between the two visits. After conducting a general analysis for the whole group, repeated analyses were done separately for participants with and without suicidal ideation (BSS = 0) at study entry. All p-values were two sided. The level of significance was set at *p* < 0.05.

## 3. Results

We recruited 103 subjects who were on antipsychotic monotherapy or polypharmacy.

In our sample 17.4% of the participants were on clozapine (*n* = 28); 3% were prescribed loxapine (*n* = 3); 26.2% were on olanzapine (*n* = 27); 3% were on fluphenazine (*n* = 3); 3% were on quetiapine (*n* = 3); 17.5% were on risperidone (*n* = 18); 4% ziprasidone (*n* = 4); 5% were on zuclopenthixol (*n* = 5); and the remaining participants were on other antipsychotics (aripiprazole, zuclopenthixol, flupenthixol, haloperidol, paliperidone, perphenazine, and pipotiazine). Overall, 21 subjects were treated with long-acting injectable antipsychotics.

Ten per cent of clozapine users were prescribed a second antipsychotic (polypharmacy). Eight percent of the subjects who were receiving long-acting injectable antipsychotics were on polypharmacy and six percent of participants using oral antipsychotics, other than clozapine, were on polypharmacy.

The patients’ clinical characteristics and demographics are described in Table 1. We did not observe suicidal behavior during the follow up and none of the 103 participants changed the primary antipsychotic during the observation time.

**Figure 1:**
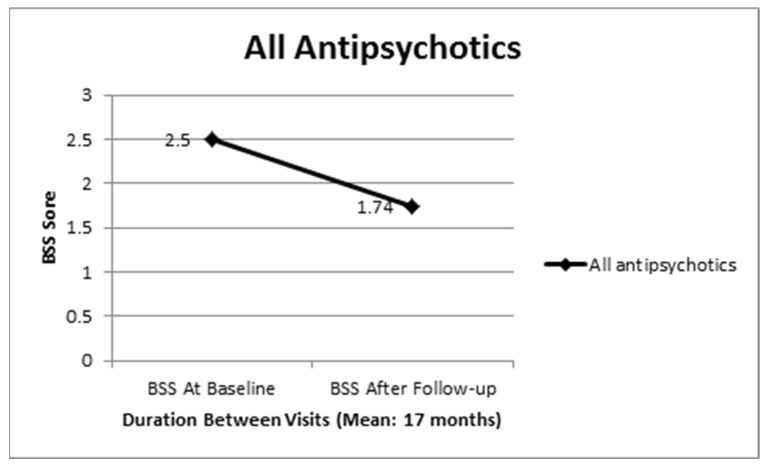
Beck Scale for Suicidal Ideation (BSS) score changes at follow-up.

Twenty-nine percent of the subjects in this study had some suicidal ideation (BSS > 0) at study entry (*n* = 30). The mean BSS score at study entry was 2.51 ± 5.4 (Figure 1). The mean interval duration between the study entry and follow-up visit was 17.4 ± 7.4 months. At follow-up, the mean BSS score was 1.74 ± 4.4. The reduction in BSS scores between the two visits was statistically significant −0.767 (95% CI: −1.5, −0.024, *p* = 0.0432). This reduction is potentially not clinically relevant but confirms the beneficial effect of a stable antipsychotic treatment in controlling suicidal ideation in psychoses.

**Figure 2:**
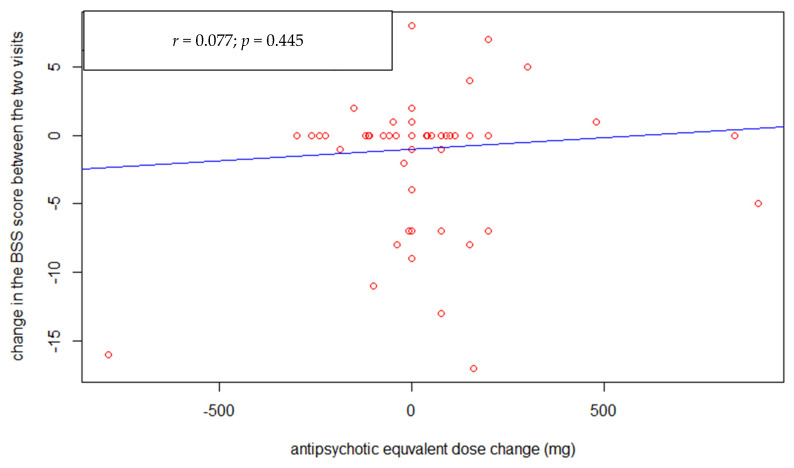
Correlation between BSS and chlorpromazine equivalent (CPZe) changes.

**Figure 3:**
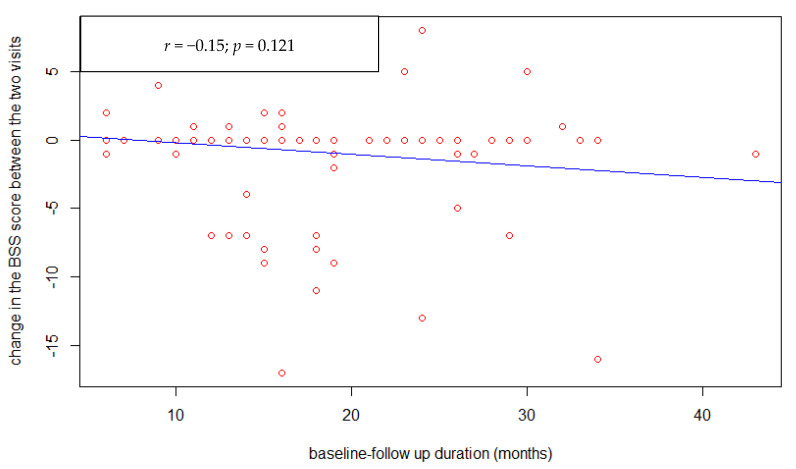
Correlation between the interval duration between the two visits and BSS change.

**Figure 4:**
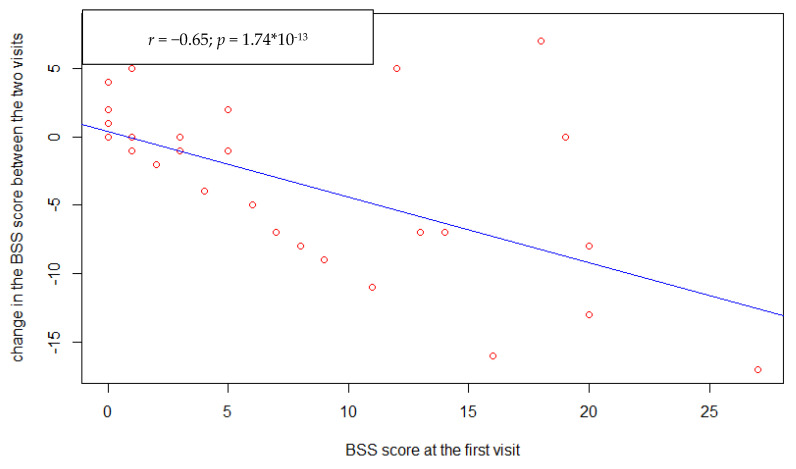
Correlation between BSS score at the first visit and its change between the two visits.

There was no correlation between changes in antipsychotics dosage (CPZe) and change in suicidal ideation severity (Figure 2) assessed by BSS (*p* = 0.44). Furthermore, there was no association between the study entry-follow-up interval duration (Figure 3) and the suicide ideation severity change (*p* = 0.12). However, there was a statistically significant negative correlation between the BSS score at study entry (Figure 4) and its change at follow-up (r = −0.65, *p* < 0.001).

### 3.1. Comparison between Clozapine and Other Antipsychotics

Subjects on clozapine (*n* = 28) were younger (40.96 ± 12.29) than the non-clozapine group (*n* = 75) (45.6 ± 13.1) but this difference was not statistically significant (*p* = 0.11). No other statistically significant differences between the two groups were found. The mean BSS score at the final visit was 1.67 ± 4.3 for the non-clozapine group and 1.93 ± 4.63 for the clozapine group. The reduction of BSS score was not significantly different between non-clozapine (−0.8) and the clozapine group (−0.93) after controlling for BSS score at study entry (*p* = 0.847). 

A separate analysis was conducted only for patients with some suicidal ideation (BSS > 0) at study entry (*n* = 30). Among this group, there were 10 participants treated with clozapine, and 20 participants treated with another antipsychotic regimen. At follow-up, there was a reduction in the overall antipsychotic dosage standardized as DDD (−0.12 ± 0.28) for the individuals treated with clozapine. In comparison, the non-clozapine users exhibited an increase of their overall dosage at follow-up (0.29 ± 0.54); however, there was no statistical difference between the two groups (*p* = 0.36). There was also a reduction in the BSS score at follow-up in both treatment groups (non-clozapine: −4.5 ± 6.71 and clozapine group: −2.80 ± 4.96), but no statistical difference was found between the two groups (*p* = 0.485).

Within patients without suicidal thoughts at study entry (n = 73), there was a trend for clozapine (*p* = 0.08) reducing the rate of emergent suicidal ideation (0.11 ± 0.47) compared to the other regimens (0.55 ± 1.6).

### 3.2. Comparison between Long-Acting Injectable and Oral Antipsychotics

The participants on oral medications (*n* = 82) including clozapine, were compared to participants on injectable antipsychotics in both monotherapy and polypharmacy (*n* = 21). Participants on oral antipsychotics were younger (42.8 ± 12.51) than participants on injectable antipsychotics (50.43 ± 13.19) (*p* = 0.015), respectively.

The individuals treated with long-acting injectable antipsychotics were less educated (*p* < 0.001), more likely to be smokers (*p* = 0.016), and more frequently treated in polypharmacy regimen (*p* = 0.004) than individuals treated only with oral antipsychotics.

Individuals treated with injectable antipsychotics showed a significant increase (*p* = 0.022) in dosage (DDD) at follow-up (+0.2 ± 0.56) compared to the individuals treated with oral antipsychotics (−0.05 ± 0.42).

The mean BSS score at follow-up was 1.56 ± 3.99 for the oral antipsychotic group and 2.43 ± 5.62 for the long-acting injectable antipsychotic group. The BSS score change at follow-up was not significantly different between individuals on oral antipsychotics (−0.93), and individuals taking long-acting injectable antipsychotics (−1.05) (*p* > 0.05).

There was no difference in BSS change between the two treatment groups when repeating the analysis for those with or without suicidal ideation at baseline.

### 3.3. Comparison between Typical and Atypical Antipsychotic Treatment

Participants on atypical antipsychotics were significantly younger (42.7 ± 12.47) than participants on typical antipsychotics 52.17 ± 12.68 (*p* = 0.017). Patients taking typical antipsychotics were more likely to smoke cigarettes (66.7%) compared to patients on atypical antipsychotics (41.2%), but this difference was not significant (*p* = 0.069). A slight increase in DDD between the two visits was observed in those on typical antipsychotics (+0.19 ± 0.41), though there was a slight decrease in the DDD for those on atypical antipsychotics (−0.02 ± 0.4). This was identified as not statistically significant (*p* = 0.072).

Both groups had reduced BSS score at follow-up (atypical antipsychotics −0.66 ± 3.81, typical antipsychotics: −1.36 ± 3.18). However, after controlling for age, baseline BSS, visit interval duration there was no difference between the effect of atypical and typical antipsychotics (*p* = 0.572).

After repeating the analysis only for those with some suicidal thoughts at study entry, there was a trend of more reduction in suicidal ideation by typical antipsychotics −7.0 ± 2.0 than atypical antipsychotics −3.6 ± 5.9 (*p* = 0.07). Though after controlling for DDD dosage change, there was no significant difference between the two antipsychotic regimens in reducing suicidal ideation (*p* = 0.568).

When analyzing only participants without suicidal ideation at study entry, there was no difference in the treatment effect between typical and atypical antipsychotics (*p* = 0.192).

### 3.4. Comparison between Antipsychotic Treatment with Antidepressants Augmentation and Antipsychotic Treatment without Antidepressants Augmentation

There were no age or sex differences between the patients treated with antidepressant and the patients treated without antidepressant augmentation. The group treated with antidepressants had higher rate of past suicide attempt (72.4%) compared to the group treated without antidepressants augmentation (34.8%) (*p* = 0.001).

At follow-up the BSS score was 1.8 ± 4.22 for the antidepressant treated group and 1.9 ± 5.02 for non-antidepressant treated group.

The BSS score change was −0.8 ± 4.02 for the group treated without antidepressants, and −0.52 ± 3.4 for the group treated with antidepressants. After controlling for BSS at study entry and interval duration between visits, there was no statistical difference between the two groups (*p* = 0.749).

Within patients with some suicidal ideation at baseline (BSS > 0), both the antidepressants and non-antidepressants treated groups had reduced BSS scores (−1.5 and −4.6, respectively). The group difference was found to not be statistically different (*p* = 0.187).

When we repeated the analysis within patients without suicidal ideation at study entry (*n* = 73), the group treated without antidepressant augmentation showed a significant worsening in suicidal ideation (0.64 ± 1.63 vs. 0 ± 0.47) compared to the group treated with antidepressant (*p* = 0.011).

## 4. Discussion

Our analysis on the pharmacological management of suicidal ideation in schizophrenia did not show a significant difference between clozapine and non-clozapine treatment, typical and atypical antipsychotics, oral and long-acting injectable medications, or antipsychotic regimen with or without antidepressants augmentation. However, these findings only apply to stable patients with schizophrenia spectrum disorders followed in outpatient settings, and not during an acute psychotic phase.

This result could imply that clozapine is not superior to the other antipsychotics in reducing suicidal ideation, replicating a previous study pertaining to the absence of a positive effect of clozapine on suicide risk in schizophrenia compared to other antipsychotics [28]. Though it is possible that a longer follow up (more than six months) is required to detect medication effect differences. Another possibility is that clozapine might have already lowered suicidal ideation by the time the study was initiated. Therefore, further research is required to confirm our findings.

It was also identified that antidepressant augmentation can protect against the development of new onset suicidal ideation in patients with schizophrenia. Our results were found to not match the result by Zisook and colleagues’ study. From their investigation, citalopram was no different than placebo in preventing “new” suicidal thoughts [21].

In this study, a few limitations were identified. The main limitation is the small sample size that has affected its statistical power. Another limitation is that we recruited patients who were already on antipsychotics at study entry. Furthermore, we did not record how long the participant was exposed to medications prior entering this study. Additionally, this study followed patients for a variable period of time. 

As per our results, age differences were influencing treatment regimen. Therefore, future studies should carefully control for age, and sex to investigate pharmacological management of suicidal ideation in schizophrenia. Additionally, we were not able to monitor adherence in the study participants. 

Lastly, our study did not consider drug interactions and pharmacogenomics influences. In fact, Spina and colleagues investigated the effect of fluoxetine on steady-state plasma concentrations of risperidone resulting in toxic levels of the latter [33], as Fluoxetine is a potent inhibitor of cytochrome P450 enzyme CYP2D6, affecting the level of several antipsychotics [33]. Therefore, future studies should consider pharmacogenomics and drug interactions to further our understanding of the effects of pharmacotherapies in treating suicidal ideation in schizophrenia.

From previous studies, there is evidence that clozapine is reducing suicidal risk in schizophrenia. As previously mentioned, these studies used strict criteria for eligible participants and the non-RCT studies might have been affected by methodological issues [28]. The authors of a meta-analysis of six RCTs evaluated clozapine treatment effectiveness on suicidal behavior, concluding that despite the significant results there were concerns regarding unmeasured clinical conditions and selection bias for participants in these studies [34].

## 5. Conclusions

This observational study investigated common psychopharmacological regimens in schizophrenia and their effectiveness in managing suicidal thoughts. We are missing high-quality information about how to approach suicidal patients with schizophrenia using pharmacotherapy. This study calls for a large simple pragmatic trial that includes a comparison between different antipsychotics in real-life samples from different clinical settings to evaluate whether antipsychotic treatment can be optimized to treat schizophrenia patients at high risk for suicide.

## Figures and Tables

**Table 1:** Clinical and Demographics of our sample (*n* = 103).

Characteristics	
Age: mean (SD)	44.4 (12.96)
Gender: male *n* (%)	68 (66)
Ethnicity: White *n* (%)	69 (67)
Marital status: married or common law *n* (%)	6 (5.8)
Employment: employed *n* (%)	38 (36.9)
Education: post-high school education *n* (%)	62 (60.2)
Smokers: *n* (%)	47 (45.6)
Lifetime drug or alcohol dependence: *n* (%)	12 (11.7)
Past suicidal attempter: *n* (%)	46 (44.7)
Average number of suicidal attempts: mean (SD)	1.1 (2.1)
Subjects experiencing a Major Depression Episode: *n* (%)	49 (47.6)
Family history of suicidal behavior: *n* (%)	20 (19.6)
Atypical antipsychotics including clozapine: *n* (%)	85 (85.9)
Clozapine users: *n* (%)	28 (27.2)
Intramuscular injection: *n* (%)	21 (20.4)
Augmenting antidepressants: *n* (%)	29 (28.2)
Polypharmacy: *n* (%)	16 (15.5)
Duration between the two visits in months: mean (SD)	17.4 (7.38)
DDD of prescribed antipsychotics on the first visit in mg: mean (SD)	1.48 (0.81)
The change of DDD between the two visits in mg: mean (SD)	−0.001 (0.459)
Participants without any changes of suicidal ideation during follow up: *n* (%)	66 (64.1)
Participants with improvement of suicidal ideation during follow up: *n* (%)	22 (21.4)
Participants with worsening of suicidal ideation during follow up: *n* (%)	15 (14.6)

## Data Availability

Data are available on request from the author. Some restrictions will apply.

## Abbreviations

BSS	Beck Scale for Suicidal Ideation
CAMH	Centre for Addiction and Mental Health
CPZe	Chlorpromazine Equivalent
DDD	Defined Daily Doses
FDA	Food and Drug Administration
RCT	Randomized Placebo-Controlled Trial
SCID	Structured Clinical Interview for DSM disorders
SD	Standard Deviation
WHO	World Health Organization
